# Aging as an active player in Alzheimer’s Disease Classification: Insights from feature selection in BrainAge Models

**DOI:** 10.1101/2025.04.16.25325953

**Published:** 2025-10-03

**Authors:** Jorge Garcia Condado, Ines Verdugo Recuero, Iñigo Tellaetxe Elorriaga, Colin Birkhenbil, Maria Carrigan, Ibai Diez, Rachel F Buckley, Asier Erramuzpe, Jesus M Cortes

**Affiliations:** a Computational Neuroimaging Lab, Biobizkaia Health Research Institute, Barakaldo, 4890, Spain; b Biomedical Research Doctorate Program, University of the Basque Country, Leioa, 48940, Spain; c Department of Neurology, Massachusetts General Hospital, Harvard Medical School, Boston, MA, 02114, USA; d Department of Signal Processing and Communications, University Carlos III of Madrid, Leganes, 28911, Spain; e Alzheimer Center Amsterdam, Neurology, Vrije Universiteit Amsterdam UMC location VUmc, Amsterdam, The Netherlands; f Amsterdam Neuroscience, Neurodegeneration, Amsterdam, The Netherlands; g Faculty of Science, Swammerdam Institute for Life Sciences, University of Amsterdam, Amsterdam, The Netherlands; h Center for Alzheimer’s Research and Treatment, Brigham & Women’s Hospital, HMS, Boston; i Melbourne School of Psychological Sciences, University of Melbourne, Melbourne, VIC, 3010, Australia; j IKERBASQUE, the Basque Foundation for Science, Bilbao, 48009, Spain; f Department of Cell Biology and Histology, University of the Basque Country, Leioa, 48940, Spain

**Keywords:** Brain Age, Alzheimer’s Disease, Neuroimaging, Neuropsychology, Machine Learning

## Abstract

**Background:**

BrainAge models estimate the biological age of the brain using neuroimaging or clinical features, making them promising tools for studying neurodegenerative diseases like Alzheimer’s disease. However, the reliance of BrainAge models on neuroimaging features such as grey matter volume and hippocampal atrophy, can introduce biases linked to individuals’ ages as these features are influenced both by normal aging and Alzheimer’s disease progression. The potential presence of such age-biases raises a critical question: can BrainAge models trained to estimate biological brain aging make meaningful contributions to Alzheimer’s diagnosis, or does any introduced age-bias conflate aging effects with disease pathology? Understanding how deliberate feature selection impacts this confounding effect is essential for developing reliable age-related biomarkers.

**Methodology:**

We ranked neuroimaging and neuropsychological features based on their mutual information with age and their discriminative power across four clinical groups: cognitively normal, Mild Cognitive Impairment, Alzheimer’s Disease, stable Mild Cognitive Impairment and progressive Mild Cognitive Impairment. Iteratively, we trained BrainAge models using different subsets of these features, some optimized for predicting aging and others for discrimination of clinical Alzheimer’s disease stages. We assess the error in BrainAge delta, the difference between predicted biological age and chronological age, and evaluate its classification performance across clinical groups. Finally, we compare using deltas for classification with a logistic regression model directly trained on the neuroimaging features used in the BrainAge models.

**Results:**

Neuroimaging features are more strongly correlated with aging, while neuropsychological features exhibit greater discriminative power for Alzheimer’s disease classification. BrainAge models optimized for age prediction yield deltas that are suboptimal when used for classifying Alzheimer’s disease, whereas models trained to generate deltas optimized to be used for classifying Alzheimer’s disease have reduced age prediction accuracy. This trade-off suggests that BrainAge models may not optimally separate aging-related changes from disease-specific alterations. BrainAge models have varying classification accuracy as compared to direct utilization of features in logistic regression. However, BrainAge provides a continuous measure, offering a single output that can be used across clinical stages, in contrast to classification approaches that require explicit labels for each disease stage.

**Conclusion:**

Aging significantly affects BrainAge-based classification of Alzheimer’s disease. Feature selection plays a critical role in mitigating this effect, as the outputs of models trained to predict age, the deltas, may fail in Alzheimer’s disease classification. These findings underscore the need for task-specific feature selection and model design to ensure that BrainAge models are appropriately applied in neurodegenerative disease research.

## 1. Introduction

BrainAge models are predictive tools developed using machine learning or deep learning techniques to estimate an individual’s chronological age based on neuroimaging data (Franke et al., 2010). BrainAge models are often employed to assess deviations from typical aging trajectories, with the difference between a participant’s predicted and actual age, referred to as the BrainAge delta, serving as a potential biomarker for various diseases. A positive delta indicates that the brain appears older than expected in reference to a healthy brain of that same age, whereas a negative delta suggests a younger-appearing brain. This metric is increasingly used as a biomarker of brain health, with higher BrainAge deltas linked to neurodegeneration and cognitive decline while lower deltas may reflect resilience to aging processes. These models have gained significant attention in the study of neurodegenerative diseases, such as Alzheimer’s disease (AD) (Gaser et al., 2013) as well as psychiatric disorders and other neurological conditions (Baecker et al., 2021).

Despite the potential of BrainAge models, several challenges hinder their generalizability and clinical utility. One critical issue is the inherent bias introduced by training models on sample populations that are not representative of the broader population. For instance, most models are developed using data from predominantly white individuals, which can result in biased predictions when applied to diverse populations (Moguilner et al., 2024; Puc et al., 2021). Additionally, the definition of “healthy” in age modeling is varies depending on the study, and this variability in training data can further skew results (de Lange et al., 2022; Franke and Gaser, 2019).

Another major challenge is the reproducibility of neuroimaging-based models, especially across different imaging sites and protocols (Korbmacher et al., 2023; Yu et al., 2024). Variability in image acquisition, preprocessing, and analysis methods can introduce noise, complicating efforts to develop models that are robust and generalizable (Gebre et al., 2023). Although initiatives like the Brain Age Standardized Evaluation (BASE) (Dular and Špiclin, 2024) aim to standardize BrainAge prediction workflows, there remains a need for consensus on evaluation criteria and the consistent application of these standards.

Our previous work has demonstrated that BrainAge models with poorer age prediction accuracy can, at times, yield deltas that perform better when used as input in downstream tasks, such as classifying between stable Mild Cognitive Impairment (sMCI) and progressive MCI (pMCI) to AD (Garcia Condado et al., 2023). This suggests that optimizing models purely for age prediction may not necessarily lead to better outcomes when using the deltas to distinguish between individuals with AD dementia and cognitively normal individuals. Despite aging being a strong predictor of dementia, it is non-specific to any one disease, making it challenging to distinguish between normal and pathological ageing.

In this work, we aim to explore how the choice of features used in BrainAge models affects both the error in age prediction and the performance of deltas when used as input in auxiliary downstream tasks, such as classifying between clinical groups. Specifically, we investigate these relationships in the context of AD to elucidate the trade-offs between prediction accuracy and classification performance. We rank neuroimaging and neuropsychological features based on their relationship with age and their discriminative power across clinical groups. By constructing BrainAge models with different feature sets, we assess the error in BrainAge delta and its classification performance when used as input to a logistic regressor. Finally, we compare the predictive value of BrainAge delta to a direct classification approach using neuroimaging features.

## 2. Materials and Methods

**Figure 1.**
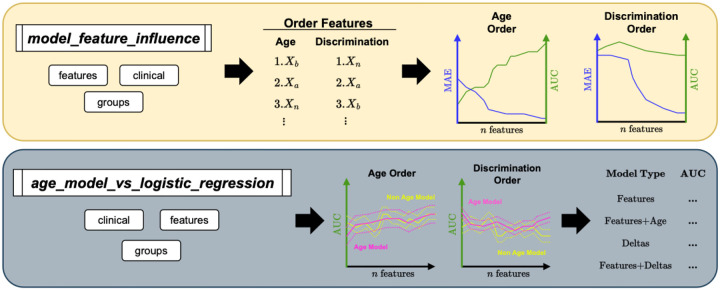
Overview of the two new commands for AgeML to explore the relationship between age prediction and classification accuracy. *Top panel:* Model Feature Influence pipeline schematic. First, the provided features are ranked according to their mutual information with age or according to their discriminative power to classify the specified clinical groups. Then, age regression and clinical classification models are trained with the computed orderings to evaluate how the progressive addition of features affects the performance of the models. The progression curves are automatically plotted. *Bottom panel:* Age Models versus direct logistic regression pipeline. The given features are first ranked according to the same criterion from above. After, two logistic regressor types are trained to classify the specified clinical groups; one based on an age delta computed from Age models; and the other directly using the features. The performance of the classifiers is plotted trained with increasingly more features added in the computed orderings, similar to the panel above. A summary table and the progression curves are automatically output.

In this study, we explore two different approaches to BrainAge modeling, see Fig. 1. Our first approach explores how BrainAge models trained on different feature sets compare in their age prediction error compared to the use of the generated deltas in the downstream task of classifying two different clinical groups. Our second approach explores whether using the BrainAge deltas for classification is better than using the features directly with a logistic regression for classification.

Both methodologies have been integrated for easy use within AgeML (Garcia Condado et al., 2025). AgeML is an OpenSource Python package for Age Modeling with Machine Learning. The first approach is integrated under the command ***model_feature_influence***, see Fig. 1 Top panel. The second approach is integrated under the command ***age_model_vs_logistic_regression***, see Fig. 1 Bottom panel. The OpenSource code can be found in the GitHub repository: github.com/compneurobilbao/ageml.

### 2.1 Data

Data used in the preparation of this article was obtained from the Alzheimer’s Disease Neuroimaging Initiative (ADNI) database (adni.loni.usc.edu) (Petersen et al., 2010). All participants in the ADNI2 and ADNI3 phases who had an initial visit with T1-weighted imaging and neuropsychological evaluation were extracted from the ADNI database. This included clinically normal older adults (CN), and MCI and AD participants. The demographics are provided in Table 1.

Using longitudinal data, we identified those who progressed from MCI to AD. sMCI participants were considered stable if they continued to be diagnosed with MCI after 3 years from the initial visit. Similarly, only participants who progressed to AD within 3 years from the initial visit were considered pMCI.

Models were built using structural brain features extracted from T1-weighted images and neuropsychological features at baseline. Ten neuroimaging volumetric features were obtained from T1-weighted images from each participant’s first visit. Three of the ten features were extracted with the Structural Image Evaluation with Normalisation of Atrophy Cross-sectional (SIENAX) (Smith et al., 2002), part of FMRIB Software Library (FSL) (Smith et al., 2004) to obtain grey matter volume, white matter volume, cerebrospinal fluid volume, as well as a volume scale value. Then the other seven features were extracted using the FMRIB’s Integrated Registration and Segmentation Tool (FIRST) (Patenaude et al., 2011) to segment and calculate the volumes of the thalamus, caudate, putamen, pallidum, hippocampus, amygdala, and accumbens over both hemispheres. The volume scale value was used to control for differences in brain size. We also extracted these same features using FreeSurfer 7.2 (Fischl, 2012) and cortical thickness measurements for the inferior parietal, inferior temporal, middle temporal, entorhinal, parahippocampal and fusiform regions. Accuracy of the segmentation tools, both sensitivity and specificity, are published elsewhere (Fischl, 2012; Patenaude et al., 2011; Smith et al., 2002). We selected features from T1-weighted imaging due to their lower variability in signal-to-noise ratio and reduced site-related differences (Warrington et al., 2025), as well as prior evidence that structural metrics outperform functional MRI measures in age modeling (Guan et al., 2023). In particular, we focused on subcortical volumes and cortical thickness because they are strongly implicated in both aging and Alzheimer’s disease such as the hippocampus (Sabuncu, 2011), amygdala (Poulin et al., 2011) and cortical thinning of the cortex (Bakkour et al., 2009).

Six neuropsychological features were obtained from neuropsychological assessments from each participant’s first visit. These consisted of scores from standard neuropsychological tests: Mini-Mental State Examination (MMSE)(Folstein et al., 1975), Alzheimer’s Disease Assessment Scale (ADAS) (“A new rating scale for Alzheimer’s disease,” 1984), Functional Assessment Questionaire (FAQ) (Pfeffer et al., 1982), and Montreal Cognitive Assessment (MoCA) (Nasreddine et al., 2005), as well as two metrics generated in the ADNI study: ADNI Memory score (for the Alzheimer’s Disease Neuroimaging Initiative et al., 2012a) and ADNI Executive Function (for the Alzheimer’s Disease Neuroimaging Initiative et al., 2012b). We compare 4 different clinical groups in the study: CN vs MCI, CN vs AD, MCI vs AD and sMCI vs pMCI.

### 2.2 Age prediction and classification accuracy for different feature sets

To create the different feature sets, we first order the features based on mutual information (MI), which is well known for capturing relationships beyond the linear dependencies detected by pairwise correlations. In particular, defined from information theory, MI quantifies the statistical dependency between variables, providing a measure of how much knowing one variable reduces uncertainty about another. We use MI to rank features according to two criteria. First, features are ordered by their MI with age using the function *mutual_information_regression* from sklearn (“Scikit-learn: Machine Learning in Python,” n.d.). This ranking reflects the extent to which each feature individually correlates with age. Second, features are ranked based on their discriminative power between two clinical groups by calculating their MI with clinical labels using the function *mutual_information_classif* from sklearn (“Scikit-learn: Machine Learning in Python,” n.d.). This approach identifies features most relevant for distinguishing between clinical labels. For each different case, feature sets are created by starting with the feature with the highest mutual information and iteratively adding the next feature with the next highest mutual information. In this study, we use 10 neuroimaging features (grey matter, white matter, cerebrospinal fluid, thalamus, caudate, putamen, pallidum, hippocampus, amygdala, and accumbens normalized volumes) and 6 neuropsychological features (MMSE, ADAS, FAQ, MoCA, ADNI Memory and ADNI executive), so a total of 16 feature sets are created.

For each feature set, we train a BrainAge model based on healthy controls. Specific details on the model training can be found in the Methods section of AgeML (Garcia Condado et al., 2025). In summary, with *y* as age, ***X*** as our feature set and *f*() as our pipeline, we optimize the parameters of our pipeline *f*() by means of minimizing the mean squared error of age on cognitively normal controls. Our pipeline consists of a feature scaler and a linear regressor and we use a 5-fold cross-validation scheme. The reported age prediction error, the Mean Absolute Error (MAE), is reported before the age bias correction step (de Lange and Cole, 2020).

Afterwards we apply the BrainAge model to the two clinical groups of interest to obtain predicted ages. We then calculate age deltas, the predicted age after age bias correction minus the chronological age, for each participant. If one of the clinical groups is the control group, the predicted ages from the cross-validation out-of-fold predictions are used. Then the deltas are used as input into a logistic regressor to classify between two groups and obtain Areas Under the Curve (AUC). To handle class imbalance within cross-validation, we first stratify the n-fold CV split to preserve the original class ratios. Within each training fold, we then apply undersampling of the majority class to match the number of participants in the smaller group. This procedure prevents information leakage whilst the test fold remains representative of the original population distribution. This avoids optimistic bias because class rebalancing was carried out separately within each cross-validation fold before computing the AUC. To address potential circularity in using cognitive test to build BrainAge models whose deltas are later used for classification, we trained the BrainAge model exclusively on healthy controls, preventing data leakage from MCI or AD subjects as argued in previous studies (Garcia Condado et al., 2023).

For each BrainAge model trained on each feature set, we obtain a MAE for the age prediction task and an AUC from using the deltas in the downstream classification task. We then plot the MAE and AUC over the number of features used in training for each model and repeat this process for the two different types of ordering.

### 2.3 Classification accuracy for different models

We also examine how classification accuracy varies across different models. Specifically, we train four logistic regression models using different inputs. Three models use BrainAge deltas as input to a logistic regressor, where the deltas are derived from three different BrainAge models: a linear regressor, a Ridge regressor, and an SVM. In addition, we train a separate logistic regression model directly on the feature sets. For this analysis, only neuroimaging features are used, as some neuropsychological test features may have been considered by clinicians when assigning clinical labels. Excluding these features helps prevent potential biases in classification when using directly the features in classification.

We are also interested in understanding the value of the BrainAge deltas in terms of whether it adds extra information to the classification task. Therefore, we also train 4 distinct logistic regressors and compare their AUC. The 4 logistic regressors use the following as input: all neuroimaging features, all neuroimaging features and the age of participants, only the BrainAge delta, all neuroimaging features and the BrainAge delta.

## 3. Results

### 3.1 Age prediction error and classification accuracy for different feature sets

**Figure 2.**
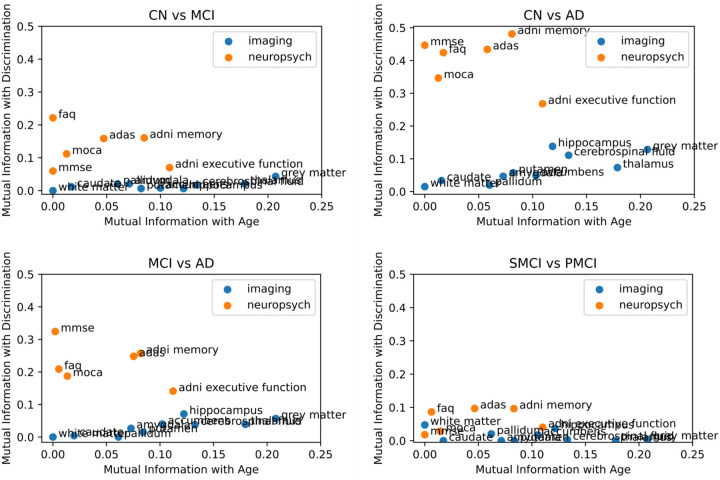
Mutual information of each feature with Age and their discriminative power. In blue are features derived from neuroimaging metrics and in orange features derived from neuropsychological testis. Control (CN), Alzheimer’s Disease (AD), Mild Cognitive Impairment (MCI), stable MCI (sMCI), and progressive MCI (pMCI)

The results of the feature ranking are shown in Table 2, listed in descending order of importance. The “Age” column refers to the relationship with age, while the remaining columns reflect the discriminatory power of each feature between clinical groups. We look at discrimination between 4 scenarios: CN vs AD, CN vs MCI, MCI vs AD and sMCI vs pMCI. Neuroimaging features were found to be more informative for inferring age, whereas neuropsychological features proved more effective in discriminating between clinical groups. When mapping MI with age and the discriminative power of each feature in Fig. 2, neuroimaging features show higher MI with age than with classification, whereas neuropsychological features show lower MI with age than with classification. However, neuropsychological features also show low MI when attempting to distinguish between sMCI and pMCI because at baseline these two subgroups are both characterized as MCI and hence, share similar cognitive profiles. Supplementary Fig. 1 shows the correlation between FIRST/FAST segmentation outputs and FreeSurfer outputs. In Supplementary Fig. 2 and Supplementary Table 1 it can be seen that cortical thickness measurements are worse at predicting age than subcortical volumes and are worse than neuropsychological features to distinguish between different groups. However, the entorhinal cortical thickness measurements show greater power at discrimination between CN and AD than neuroimaging features of subcortical volumes. Many of the cortical thickness measurements are also more powerful at discriminating sMCI vs pMCI than subcortical volumes.

**Figure. 3.**
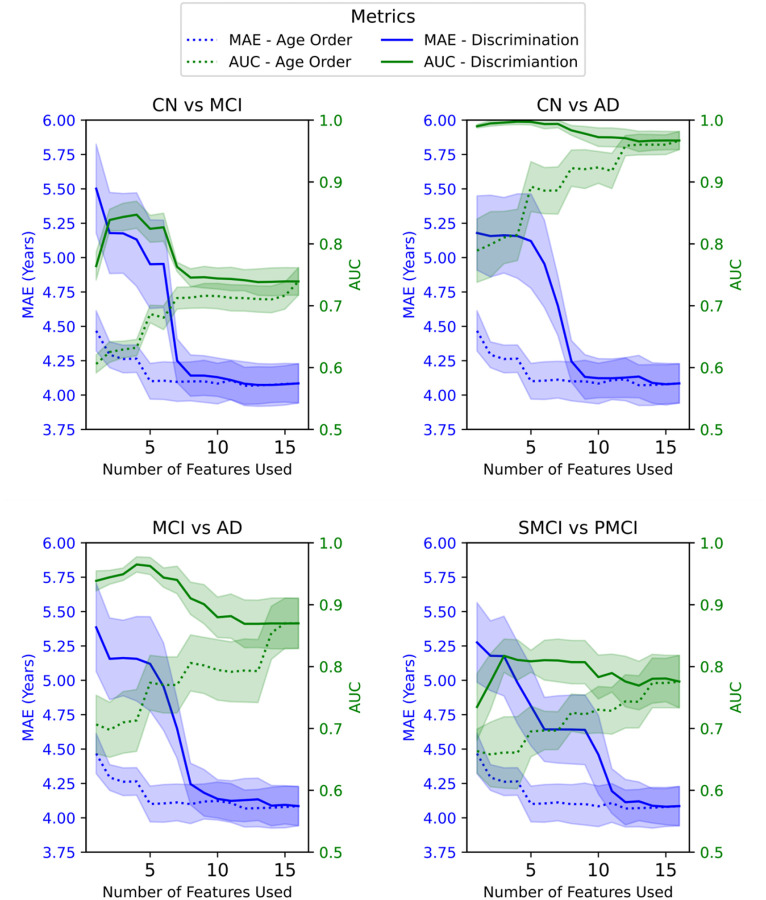
Comparison of the performance metrics, Mean Absolute Error (MAE), and Area Under the Curve (AUC) for different health condition groups using BrainAge Modelling. Features are added to the BrainAge model in descending order based on their age relationship (dotted line). Additionally, features are added to the BrainAge model in descending order according to their importance in discriminating between the following groups: Control (CN), Alzheimer’s Disease (AD), Mild Cognitive Impairment (MCI), stable MCI (sMCI), and progressive MCI (pMCI) (solid line). The shaded areas show the 95% confidence intervals of the MAE and AUC measurements.

After obtaining the different feature rankings, the pipeline is executed four times, once for each of the four classification tasks of interest: CN vs AD, CN vs MCI, MCI vs AD and sMCI vs pMCI. Results are shown in Fig. 3. This figure illustrates how MAE and AUC evolve as more features are added to each set, depending on whether the features are more relevant for age modeling or for classifying clinical groups. When features are ranked by their relevance to age, the initial models trained on the top-ranked subset tend to predict age more accurately, but the deltas perform worse in classification tasks. We see the opposite effect when the features are ranked in importance to discriminate. However, when classifying sMCI and pMCI, we observe that the age-based and discrimination-based models begin to overlap earlier as more features are added because neuropsychological features show MI values comparable to those of neuroimaging features in distinguishing between these two groups. In Supplementary Fig. 3 we see that adding cortical thickness measurements yields similar results.

### 3.2 Logistic Regression using features vs BrainAge deltas

**Figure. 4.**
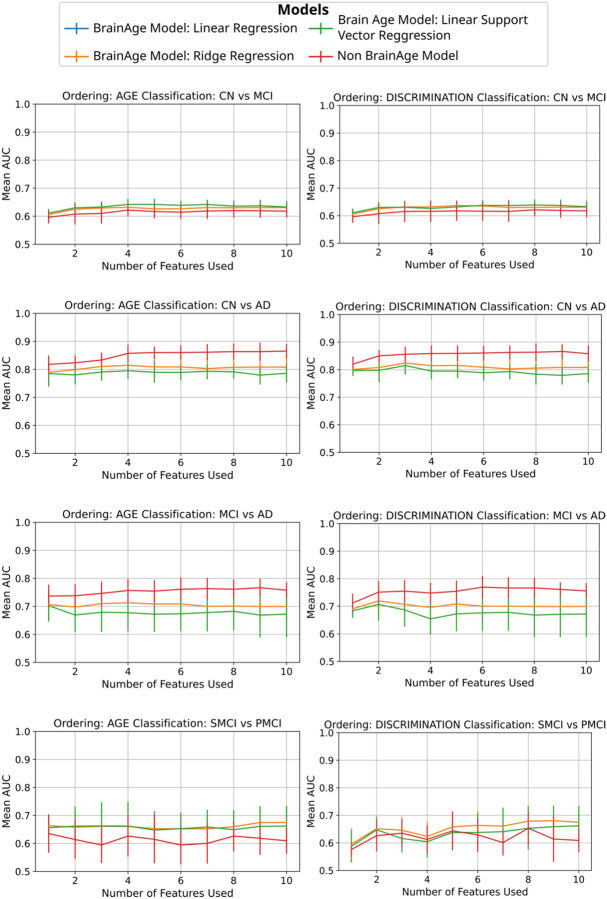
Comparison of the Area Under the Curve (AUC) using different machine learning models for classification using neuroimaging features. *Blue line:* Input to logistic regressor: Delta. BrainAge model: linear regression. *Orange line:* Input to logistic regressor: Delta. BrainAge model: Ridge. *Green line:* Input to logistic regressor: Delta. BrainAge model: Support Vector Regressor. *Red line:* Input to logistic regressor: Neuroimaging Features, No BrainAge modeling. Models are tested and trained across clinical classification groups and features ordering by Age Relationship and by discrimination between groups order (Control Group (CN), Alzheimer Disease (AD), Mild Cognitive Impairment (MCI), stable Mild Cognitive Impairment (sMCI) and progressive Mild Cognitive Impairment (pMCI)). The blue and orange line overlap. Error bars show the standard deviation of the AUC across CV folds.

We trained three different BrainAge models: a linear regressor, a ridge regressor and a SVM with different neuroimaging features. We compared the classification performance of BrainAge deltas with that of using the original features directly in a logistic regressor. The results for all four scenarios --CN vs AD, CN vs MCI, MCI vs AD and sMCI vs pMCI-- are shown in Fig. 4. Using the original features as input to the logistic regressor yielded the best performance in CN vs AD and CN vs MCI, similar performance to the other models in MCI vs AD but worse in sMCI vs pMCI. In Supplementary Fig. 4 we see that adding cortical thickness measurements which have higher power of discrimination cause the AUC performance of the logistic regressor trained on the original features to outperform all BrainAge models, that do not see an increase in AUC performance.

Finally, using all neuroimaging features, we evaluated whether adding age or the delta as an additional feature could improve classification performance. The results for each scenario are summarized in Table 3. Adding neither age nor the delta appeared to improve the AUC, as most values remained within one standard deviation of each other. Repeating the experiment with 5 different random seeds did not yield fluctuating results as seen in the standard deviations of Supplementary Table 2.

## 4. Discussion

This study aimed to evaluate how different neuroimaging and neuropsychological features influence BrainAge predictions and the utility of BrainAge deltas in classifying AD participants. By analyzing the relationship between these features and both age estimation and clinical group distinctions, we assessed the reliability of BrainAge deltas as biomarkers. Our findings provide insight into the strengths and limitations of using BrainAge models for AD classification, highlighting key factors that impact their performance and the need for careful feature selection based on specific research and clinical objectives.

As expected, ranking features by their age prediction accuracy and discriminative power highlights the distinct contributions of neuroimaging and neuropsychological features. Neuroimaging features, specifically grey matter and particularly those related to brain structures like the hippocampus, thalamus, and amygdala, are highly effective in predicting age. In contrast, neuropsychological scores excel at discriminating between clinical groups. This supports the idea that age prediction relies more on the structural integrity of brain regions, while cognitive performance metrics are more sensitive to the pathological distinctions between clinical groups.

It is important to consider possible circularity in using BrainAge deltas built with neuropsychological testing features for classification due to their role in clinical diagnosis, i.e., assigning healthy control, MCI, and AD labels in the first place. In ADNI for example, diagnostic assessment of AD staging includes a selection of these tests in combination with cutoff points and other clinical assessments, such as the Clincal Dementia Rating score not used in this study (Petersen et al., 2010). In the present study, this concern was accounted for by training our BrainAge model on healthy controls and, and thus preventing data leakage in terms of biases in the BrainAge deltas, as the model is not trained on MCI or AD. In the specific case of classifying sMCI and pMCI, despite neuropsychological tests contributing to initial diagnostic classification, our approach avoids circularity by use of baseline scores to predict future conversion rather than reclassifying subjects based on later assessments (Garcia Condado et al., 2023). Excluding neuropsychological features reduced sMCI vs. pMCI classification performance from 0.91 to 0.68 AUC (Garcia Condado et al., 2023). In Fig. 3, this is further illustrated at x = 1, where the full green line is always a neuropsychological feature and achieves a substantially higher AUC than the dotted green line which is a single neuroimaging feature. These findings indicate that neuropsychological tests carry predictive information beyond their traditional role in diagnostic labeling based on cutoffs, supporting their validity for assessing and monitoring disease progression. We acknowledge a potential limitation and bias in using neuropsychological tests that were part of the clinical decision-making process. Further research is needed using more deeply phenotyped cohorts to explore the effect of training on different neuropsychological tests that were not used in the diagnosis labelling process.

Notably, the hippocampus is consistently identified for its high discriminative power across all clinical groups. The hippocampus is among the earliest regions affected by AD pathology, and its atrophy is strongly associated to memory deficits, a hallmark of the disease. In the discrimination tasks, hippocampal volumes are effective at distinguishing between CN, MCI, and AD groups, which aligns with the well-established literature on the role of the hippocampus in cognitive decline (Mu and Gage, 2011). The early inclusion of hippocampal features in both age and discrimination-ordered sets further underscores its dual importance in aging and AD progression. This is consistent with previous findings showing that hippocampal connectivity is indeed significantly affected by aging, but, other circuits—such as the fronto-striato-thalamic network—are even more severely disrupted (see Fig. S4 in Bonifazi et al) (Bonifazi et al., 2018). Thus, although the hippocampus is involved in both aging and AD, its prominent role in AD disease may reflect a stronger association with pathological processes rather than normal aging, highlighting the interaction between these two mechanisms.

Our results show clear distinctions between BrainAge models trained on feature sets ranked by age than those ranked by their discriminative power across clinical groups. When features are ranked based on their relationship with age, the MAE of BrainAge models begin at a lower value, indicating that the model achieves higher age prediction accuracy with the top-ranked features. In this case, when using the produced delta for classification the AUC increases steadily as more features are included in the BrainAge model. Thus, reflecting progressively improved ability of the generated deltas to discriminate between clinical groups. This suggests that age-related features provide a solid foundation for accurate age prediction, while classification performance improves progressively as additional, more diverse features are incorporated.

Conversely, when features are ranked by their discriminative power, the BrainAge model’s delta AUC shows a higher intercept, reflecting better initial classification performance. However, the MAE of the BrainAge model has a higher intercept compared to the age-ranked feature sets, indicating less accurate age predictions at the onset. Hence, better age prediction does not always mean better classification power. A key transition point occurs when neuroimaging features start to appear in the discrimination-ordered set, after which the AUC stabilizes and MAE decreases. However, we do not see an increase in AUC. This indicates that neuroimaging features enhance age prediction but worsen classification accuracy.

Our previous study showed that models just trained on neuropsychological data outperformed those trained on neuroimaging data on the classification task but not in age prediction error (Garcia Condado et al., 2023). We acknowledge that this is to be expected since neuropsychological testing is used to diagnose and is an inherent part of the process. However, as argued above this is not circular reasoning since the BrainAge models are trained in the healthy cohort and never use neuropsychological data from MCI or AD during training. Hence, it is important to determine what the use of the calculated deltas will be when creating BrainAge models. Subsequently, the model should be trained with the features required for its respective task.

Recent studies have emphasized the importance of distinguishing between brain changes due to typical aging and those caused by AD. Hwang et al. (2022) introduced machine learning models such as SPARE-AD and SPARE-BA to decouple the effects of aging and neurodegeneration in brain imaging data (Hwang et al., 2022). Their results showed that by employing conservative molecular diagnoses and introducing Alzheimer’s continuum cases, it was possible to derive more specific neuroanatomical biomarkers for aging and AD, reducing the overlap in brain regions affected by both processes. Similarly, Binette et al. (2020) investigated grey matter differences across a wide age range and in AD patients, finding that while both aging and AD contribute to widespread brain atrophy, AD uniquely disrupts whole-brain morphometric organization (Pichet Binette et al., 2020). This disruption in grey matter pattern, more than volume loss, was strongly associated with cognitive impairment, highlighting the need to account for these distinct mechanisms when assessing BrainAge. Alternatively, feature importance for biological aging FIBA has been proposed as a method to improve the relevance of age models by identifying features that contribute specifically to biological rather than chronological age, refining their association with cognitive traits (Oh et al., 2023).

Our results indicate that training a logistic regression directly on the features either outperforms or equals a logistic regression trained on BrainAge deltas. The results tend to be similar or worse when the classification task is harder, like when distinguishing sMCI vs pMCI. However, when cortical thickness measurements are included, BrainAge models perform worse at distinguishing between sMCI vs pMCI. While a direct classifier can exploit the discriminative value of cortical thickness, this information appears to be lost in the BrainAge modelling process. This is in line with a recent study that found that neural network-based BrainAge models, when retrained for classification, perform worse than models trained directly for classification (Tan et al., 2025). Training on different regression models, such as linear regression ridge regression, and SVM does not improve performance. This outcome is likely influenced by the high linear correlation among neuroimaging features, as a model using a single key feature often performs similarly to those incorporating multiple features. Grey matter volume in particular plays a dominant role in both age prediction and discrimination across clinical groups, highlighting its central importance in distinguishing disease states.

However, BrainAge offers an advantage over training a logistic regressor directly with features. BrainAge deltas encapsulate disease progression within a single metric rather than requiring training separate classification models for each clinical group. Prior studies have shown that BrainAge deltas correlate with the time to conversion from MCI to AD (Garcia Condado et al., 2023), making them a potentially useful quantitative biomarker for assessing risk. This further underscores the need for careful consideration when applying BrainAge models to clinical decision-making.

Adding age or BrainAge delta as additional features does not consistently improve classification accuracy, as indicated by minimal AUC increases that remain within standard deviations. This variability suggests that neither age nor delta provides substantial independent discriminative value beyond the primary neuroimaging features. Notably, repeating the classification process with different data partitions and cross-validation folds leads to wide fluctuations in results, emphasizing the sensitivity of the models to sample variation. This finding points to the need for further research to better understand the potential utility of age and delta features in classification, as well as the robustness of these models across varying sample conditions. Recent studies suggest that using chronological age as a pretraining target might be suboptimal for predicting specific health outcomes (Tan et al., 2025).

A limitation of this work is that only neuroimaging features from a single modality, T1-weighted images, are included in the analysis. Features from these modalities were chosen because there is lower variability compared to diffusion or functional brain imaging (Warrington et al., 2025). While the ADNI dataset offers rich multimodal imaging, it was collected across a large number of scanners with relatively few subjects per site (e.g., fewer than 10 participants in many centers), which introduces substantial site-related variability. This effect is particularly problematic for modalities such as diffusion MRI and functional MRI, where sequence parameters, scanner hardware, and acquisition protocols strongly influence signal-to-noise ratios and overall reproducibility. Previous studies have also shown that structural based metrics outperform functional MRI metrics at the age modelling prediction task (Guan et al., 2023). Including more quantitative measurements such as cortical thickness measurements did not improve age prediction accuracies in line with previous studies (Guan et al., 2023). On the other hand, the processing pipelines we have employed are fully open-source, and we encourage further exploration of multimodal approaches using datasets that are more homogeneous or specifically designed to minimize inter-site variability.

## Conclusion

Our findings demonstrate that neuroimaging and neuropsychological features play distinct roles in age prediction and disease classification in the context of BrainAge modeling, with neuroimaging features excelling in age prediction and neuropsychological features showing greater sensitivity to clinical distinctions. The hippocampus and grey matter volume emerge as critical biomarkers in both aging and Alzheimer’s disease progression. The instability of results across different model configurations and data splits suggests a need for further exploration into feature robustness and optimal model selection based on task-specific requirements. Overall, while BrainAge deltas can provide a useful single metric associated with the risk of developing Alzheimer’s disease, their application should be carefully considered in the context of specific clinical and research goals.

## Supplementary Material

1

## Figures and Tables

**Table 1.** Cohort demographics

Characteristic	CN N = 629^1^	MCI N = 635^1^	AD N = 208^1^	sMCI N = 238^1^	pMCI N = 98^1^	p-value^2^
Gender						<0.001
Female	364 (58%)	279 (44%)	87 (42%)	110 (46%)	43 (44%)	
Male	265 (42%)	356 (56%)	121 (58%)	128 (54%)	55 (56%)	
Age (Years)	72 (7)	72 (8)	75 (8)	73 (8)	73 (7)	<0.001
Education (Years)	17 (2)	16 (3)	16 (3)	16 (3)	16 (3)	<0.001

1 n (%); Mean (SD)

2 Pearson’s Chi-squared test; Kruskal-Wallis rank sum test

**Table 2.** Results of ranking features across different orderings. The features are ordered in descending importance according to the variable indicated in the column header. The first column indicates ordering according to mutual information with age, while the subsequent columns make a comparison between the mutual information of features for the following groups: Control Group (CN), Alzheimer’s Diseases (AD), Mild Cognitive Impairment (MCI), Stable Mild Cognitive Impairment (sMCI) and Progressive Mild Cognitive Impairment (pMCI).

Set	Age	CN vs AD	CN vs MCI	MCI vs AD	sMCI vs pMCI
1	Grey Matter	ADNI Memory	ADNI Memory	MMSE	ADAS
2	Thalamus	MMSE	FAQ	ADAS	ADNI Memory
3	Cerebrospinal Fluid	FAQ	ADAS	ADNI Memory	MMSE
4	Hippocampus	ADAS	MMSE	FAQ	FAQ
5	ADNI Executive Function	MoCA	MoCA	MoCA	White Matter
6	Accumbens	ADNI Executive Function	ADNI Executive Function	ADNI Executive Function	MoCA
7	ADNI Memory	Hippocampus	Grey Matter	Hippocampus	ADNI Executive Function
8	Putamen	Grey Matter	Thalamus	Grey Matter	Hippocampus
9	Amygdala	Cerebrospinal Fluid	Amygdala	Accumbens	Pallidum
10	Pallidum	Thalamus	Pallidum	Thalamus	Accumbens
11	ADAS	Putamen	Cerebrospinal Fluid	Cerebrospinal Fluid	Grey Matter
12	Caudate	Accumbens	Caudate	Amygdala	Cerebrospinal Fluid
13	MoCA	Amygdala	Accumbens	Putamen	Thalamus
14	FAQ	Caudate	Putamen	Caudate	Caudate
15	White Matter	Pallidum	Hippocampus	White Matter	Putamen
16	MMSE	White Matter	White Matter	Pallidum	Amygdala

**Table 3.** Comparison of the Area Under the Curve (AUC) across various clinical classifications using different input feature sets. The classification was performed using a Logistic Regressor and included all brain structural features for the following groups: Control Group (CN), Alzheimer’s Disease (AD), Mild Cognitive Impairment (MCI), stable Mild Cognitive Impairment (sMCI), and progressive Mild Cognitive Impairment (pMCI). ± indicates standard deviations across CV folds.

Groups	Features	Features + Age	Delta	Features + Delta
CN vs MCI	0.62 ± 0.02	0.63 ± 0.02	0.63 ± 0.03	0.63 ± 0.02
CN vs AD	0.86 ± 0.03	0.87 ± 0.03	0.81 ± 0.04	0.87 ± 0.03
MCI vs AD	0.76 ± 0.03	0.76 ± 0.03	0.69 ± 0.05	0.76 ± 0.04
pMCI vs sMCI	0.58 ± 0.09	0.62 ± 0.07	0.67 ± 0.04	0.63 ± 0.07

## Data Availability

The software used in this work is open source and publicly available: www.github.com/compneurobilbao/ageml.
