# Supplementary material for: Aging as an active player in Alzheimer’s Disease Classification: Insights from feature selection in BrainAge Models
Source: medRxiv. 2025 Oct 3:2025.04.16.25325953. Preprint. [Version 2] doi: 10.1101/2025.04.16.25325953 (PMC12622099; doi:10.1101/2025.04.16.25325953)
Supplement: 1 [file NIHPP2025.04.16.25325953V2-supplement-1.pdf]

medRxiv preprint doi: https://doi.org/10.1101/2025.04.16.25325953; this version posted October 3, 2025. The copyright holder for this preprint (which was not certified by peer review) is the author/funder, who has granted medRxiv a license to display the preprint in perpetuity.
It is made available under a CC-BY 4.0 International license .

Supplementary Figures

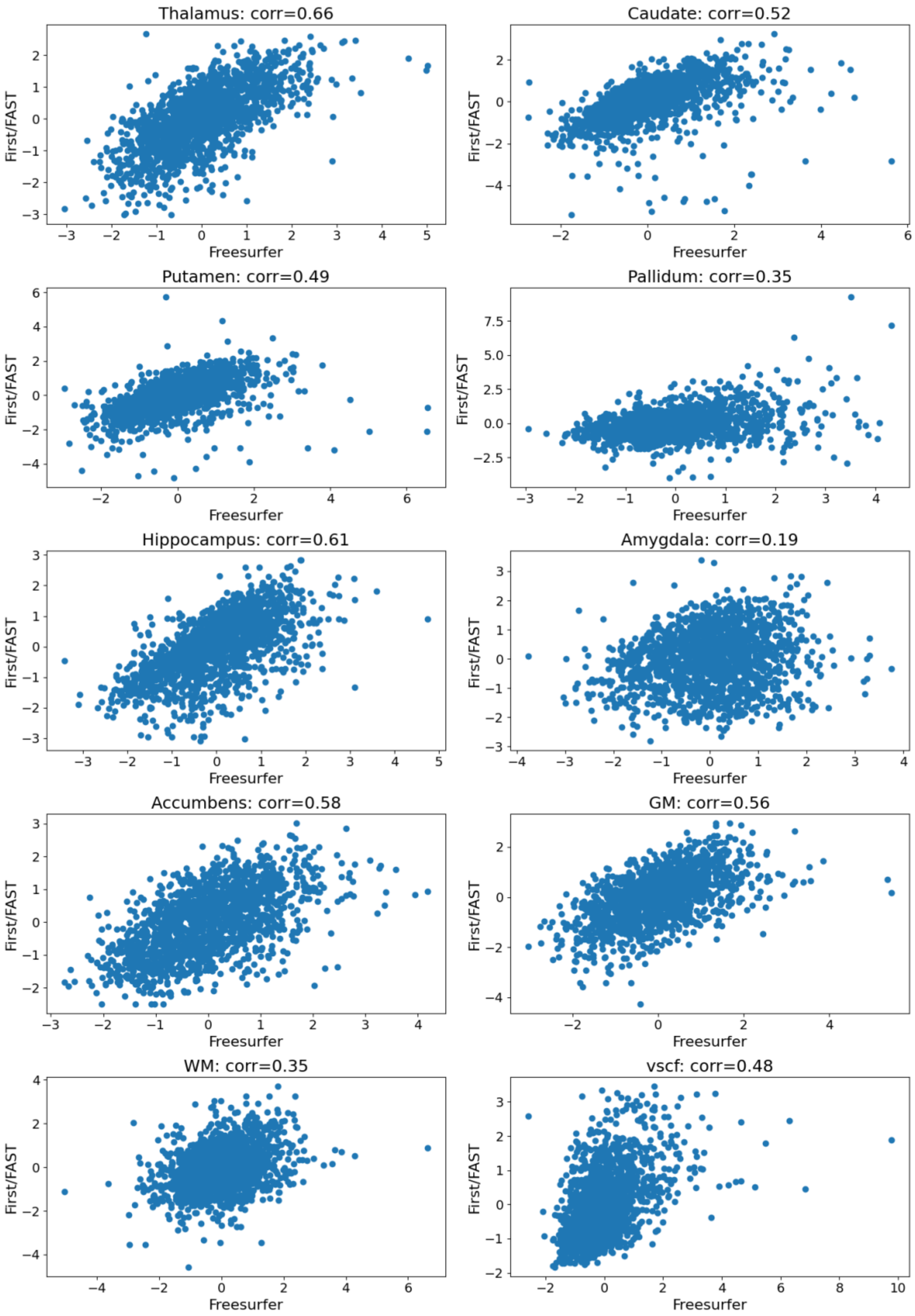

medRxiv preprint doi: https://doi.org/10.1101/2025.04.16.25325953; this version posted October 3, 2025. The copyright holder for this preprint (which was not certified by peer review) is the author/funder, who has granted medRxiv a license to display the preprint in perpetuity.
It is made available under a CC-BY 4.0 International license .

Supplementary Figure 1. Normalized values for FIRST/FAST segmentation of cortical and subcortical volumes in comparison to normalized values of FreeSurfer Segmentation. Values shown are Pearson correlation values.

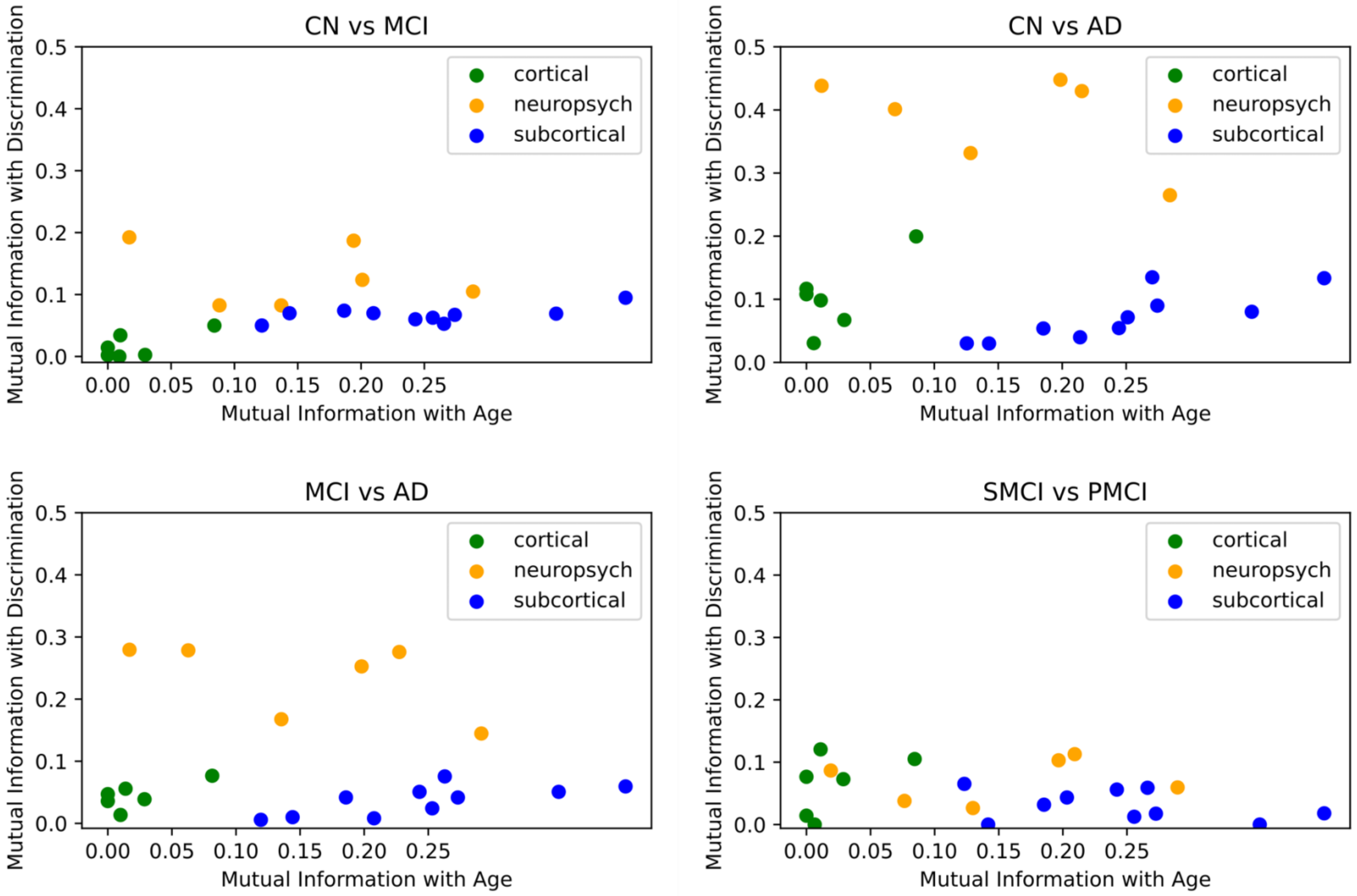
CN vs MCI
CN vs AD
MCI vs AD
SMCI vs PMCI
cortical
neuropsych
subcortical
Mutual Information with Discrimination
Mutual Information with Age
0.0
0.1
0.2
0.3
0.4
0.5
0.00
0.05
0.10
0.15
0.20
0.25

Supplementary Figure 2. Mutual information of each feature with Age and their discriminative power. In blue are features derived from neuroimaging metrics of subcortical volumes using FIRST/FAST, in orange features derived from neuropsychological tests and in green neuroimaging metrics of cortical thickness using FreeSurfer. Control (CN), Alzheimer's Disease (AD), Mild Cognitive Impairment (MCI), stable MCI (sMCI), and progressive MCI (pMCI). Each specific feature name in the graph can be derived from Supplementary Table 1.

medRxiv preprint doi: https://doi.org/10.1101/2025.04.16.25325953; this version posted October 3, 2025. The copyright holder for this preprint (which was not certified by peer review) is the author/funder, who has granted medRxiv a license to display the preprint in perpetuity.
It is made available under a CC-BY 4.0 International license .

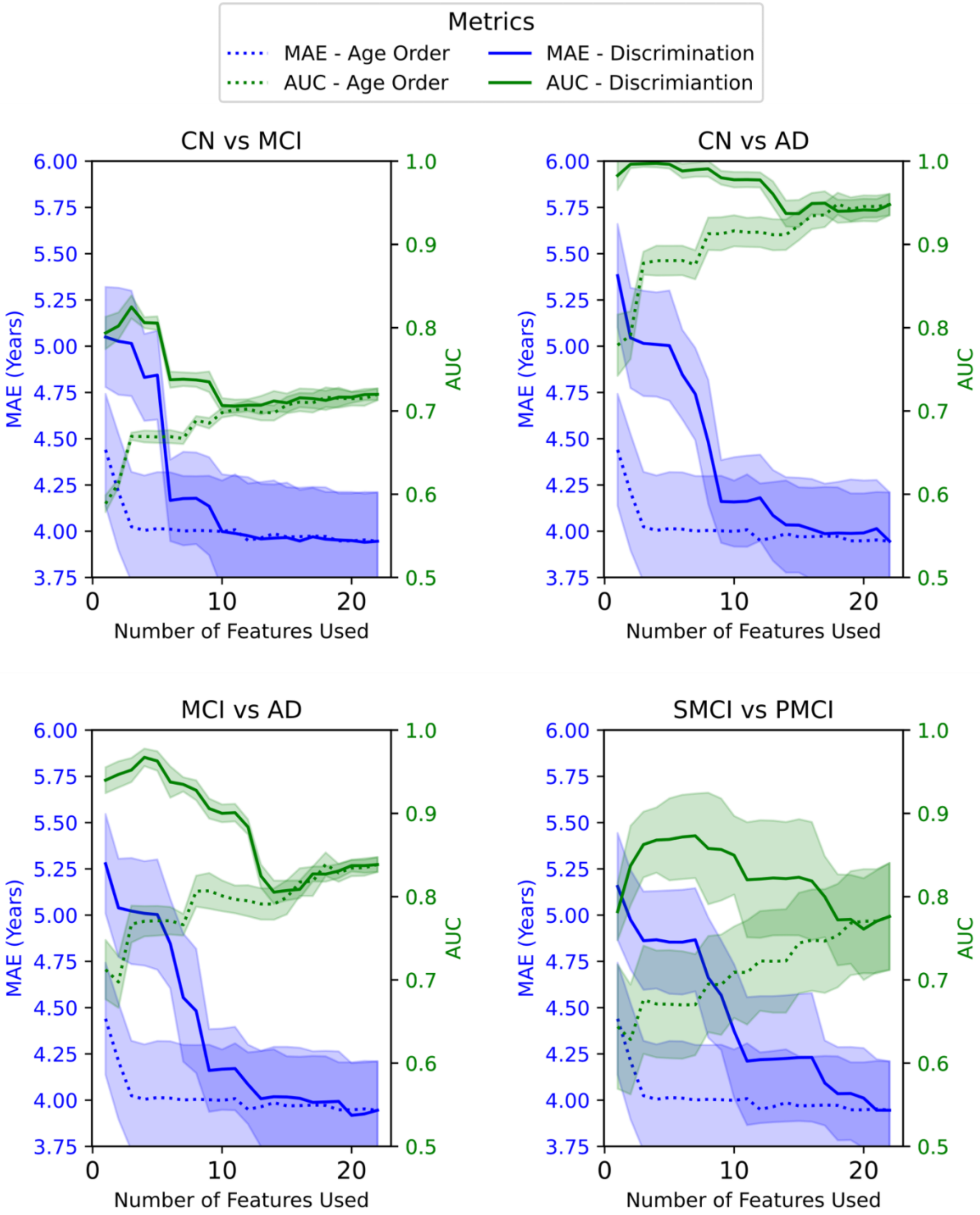

Supplementary Figure. 3. Comparison of the performance metrics, Mean Absolute Error (MAE), and Area Under the Curve (AUC) for different health condition groups using

medRxiv preprint doi: https://doi.org/10.1101/2025.04.16.25325953; this version posted October 3, 2025. The copyright holder for this preprint (which was not certified by peer review) is the author/funder, who has granted medRxiv a license to display the preprint in perpetuity.
It is made available under a CC-BY 4.0 International license .

BrainAge Modelling including cortical thickness measurements. Features are added to the BrainAge model in descending order based on their age relationship (dotted line). Additionally, features are added to the BrainAge model in descending order according to their importance in discriminating between the following groups: Control (CN), Alzheimer's Disease (AD), Mild Cognitive Impairment (MCI), stable MCI (sMCI), and progressive MCI (pMCI) (solid line). The shaded areas show the 95% confidence intervals of the MAE and AUC measurements.

medRxiv preprint doi: https://doi.org/10.1101/2025.04.16.25325953; this version posted October 3, 2025. The copyright holder for this preprint (which was not certified by peer review) is the author/funder, who has granted medRxiv a license to display the preprint in perpetuity.
It is made available under a CC-BY 4.0 International license .

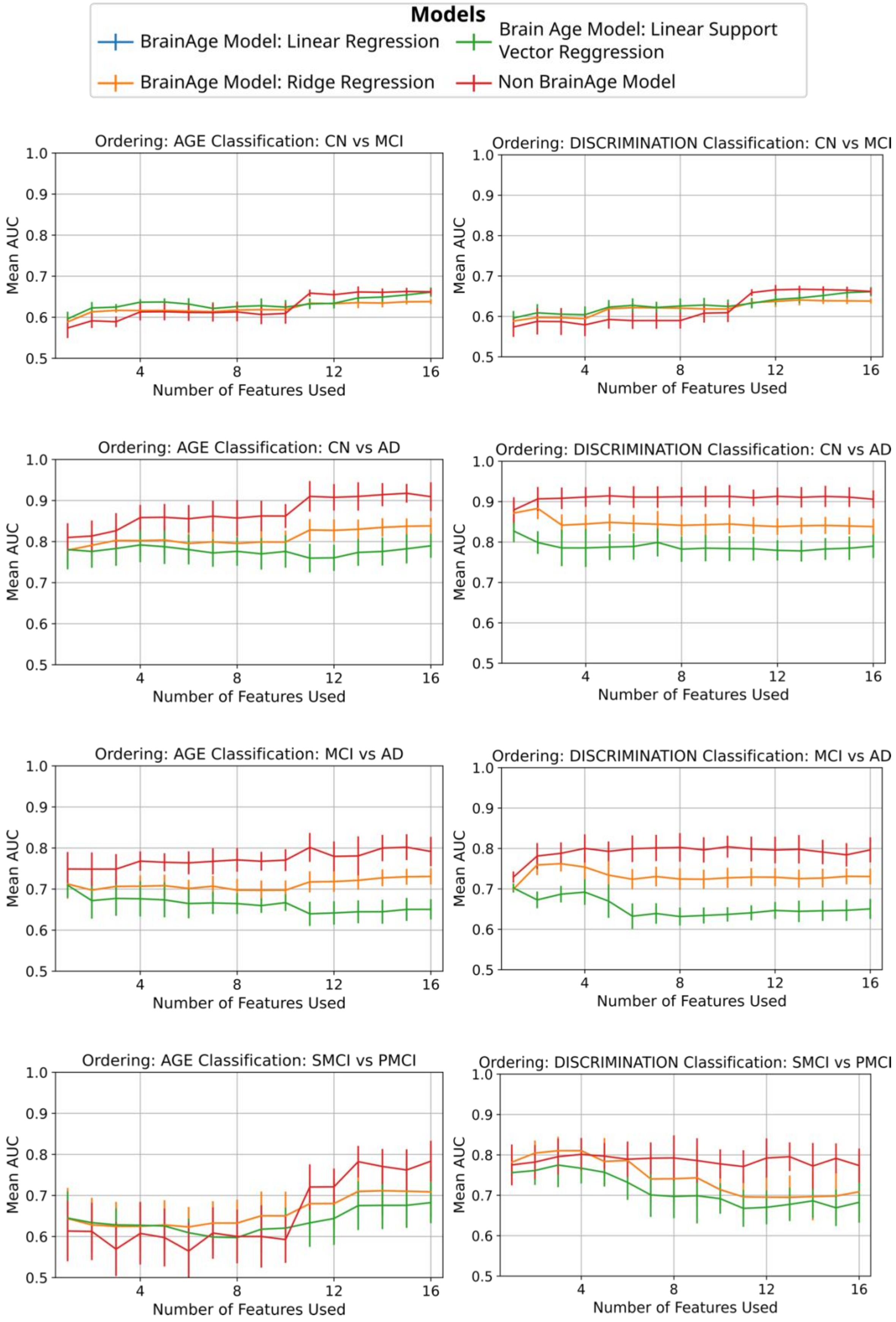

medRxiv preprint doi: https://doi.org/10.1101/2025.04.16.25325953; this version posted October 3, 2025. The copyright holder for this preprint (which was not certified by peer review) is the author/funder, who has granted medRxiv a license to display the preprint in perpetuity.
It is made available under a CC-BY 4.0 International license .

Figure. 4. Comparison of the Area Under the Curve (AUC) using different machine learning models for classification using neuroimaging features including cortical thickness measurements. *Blue line:* Input to logistic regressor: Delta. BrainAge model: linear regression. *Orange line:* Input to logistic regressor: Delta. BrainAge model: Ridge. *Green line:* Input to logistic regressor: Delta. BrainAge model: Support Vector Regressor. *Red line:* Input to logistic regressor: Neuroimaging Features, No BrainAge modeling. Models are tested and trained across clinical classification groups and features ordering by Age Relationship and by discrimination between groups order (Control Group (CN), Alzheimer Disease (AD), Mild Cognitive Impairment (MCI), stable Mild Cognitive Impairment (sMCI) and progressive Mild Cognitive Impairment (pMCI)). The blue and orange line overlap Error bars show the standard deviation of the AUC across CV folds.

medRxiv preprint doi: https://doi.org/10.1101/2025.04.16.25325953; this version posted October 3, 2025. The copyright holder for this preprint (which was not certified by peer review) is the author/funder, who has granted medRxiv a license to display the preprint in perpetuity.
It is made available under a CC-BY 4.0 International license .

Supplementary Tables

Table 1. Results of ranking features across different orderings including neuroimaging volumetric features, neuroimaging cortical thickness features and neuropsychological features. The features are ordered in descending importance according to the variable indicated in the column header. The first column indicates ordering according to mutual information with age, while the subsequent columns make a comparison between the mutual information of features for the following groups: Control Group (CN), Alzheimer's Diseases (AD), Mild Cognitive Impairment (MCI), Stable Mild Cognitive Impairment (sMCI) and Progressive Mild Cognitive Impairment (pMCI).

Set	Age	CN vs AD	CN vs MCI	MCI vs AD	sMCI vs pMCI
1	Grey Matter	FAQ	ADNI Memory	MMSE	Fusiform
2	Thalamus	ADNI Memory	ADAS	ADNI Memory	ADAS
3	ADNI Executive Function	ADAS	FAQ	ADAS	ADNI Memory
4	Cerebrospinal Fluid	MMSE	ADNI Executive Function	FAQ	FAQ
5	Hippocampus	MoCA	MoCA	MoCA	Entorhinal
6	Putamen	ADNI Executive Function	Grey Matter	ADNI Executive Function	Parahippocampal
7	Accumbens	Entorhinal	Amygdala	Hippocampus	Inferior temporal
8	ADAS	Hippocampus	Pallidum	Entorhinal	White Matter

medRxiv preprint doi: https://doi.org/10.1101/2025.04.16.25325953; this version posted October 3, 2025. The copyright holder for this preprint (which was not certified by peer review) is the author/funder, who has granted medRxiv a license to display the preprint in perpetuity.
It is made available under a CC-BY 4.0 International license .

9	Pallidum	Grey Matter	Caudate	Grey Matter	ADNI Executive Function
10	ADNI Memory	Inferior temporal	Thalamus	Fusiform	Hippocampus
11	Amygdala	Middle temporal	Cerebrospinal Fluid	Middle temporal	Accumbens
12	Caudate	Fusiform	Putamen	Accumbens	Pallidum
13	MoCA	Cerebrospinal Fluid	Accumbens	Thalamus	MMSE
14	White Matter	Thalamus	MMSE	Amygdala	MoCA
15	Entorhinal	Putamen	Hippocampus	Cerebrospinal Fluid	Amygdala
16	MMSE	Parahippocampal	Entorhinal	Inferior temporal	Middle temporal
17	Parahippocampal	Accumbens	White Matter	Parahippocampal	Grey Matter
18	FAQ	Amygdala	Fusiform	Putamen	Cerebrospinal Fluid
19	Fusiform	Pallidum	Inferior temporal	Inferior parietal	Putamen
20	Inferior parietal	Inferior parietal	Inferior parietal	Caudate	Thalamus
21	Middle temporal	White Matter	Parahippocampal	Pallidum	Caudate
22	Inferior temporal	Caudate	Middle temporal	White Matter	Inferiorparietal

medRxiv preprint doi: https://doi.org/10.1101/2025.04.16.25325953; this version posted October 3, 2025. The copyright holder for this preprint (which was not certified by peer review) is the author/funder, who has granted medRxiv a license to display the preprint in perpetuity.
It is made available under a CC-BY 4.0 International license .

Supplementary Table 2. Comparison of the Area Under the Curve (AUC) across various clinical classifications using different input feature sets and 5 different random seeds. The classification was performed using a Logistic Regressor and included all brain structural features for the following groups: Control Group (CN), Alzheimer's Disease (AD), Mild Cognitive Impairment (MCI), stable Mild Cognitive Impairment (sMCI), and progressive Mild Cognitive Impairment (pMCI). ± indicates standard deviations across seeds.

Groups	Features	Features + Age	Delta	Features + Delta
CN vs MCI	0.62 ± 10^{-4}	0.63 ± 10^{-3}	0.63 ± 10^{-7}	0.63 ± 10^{-4}
CN vs AD	0.86 ± 10^{-3}	0.86 ± 10^{-3}	0.80 ± 10^{-8}	0.86 ± 10^{-3}
MCI vs AD	0.76 ± 10^{-3}	0.76 ± 10^{-3}	0.69 ± 10^{-7}	0.76 ± 10^{-3}
pMCI vs sMCI	0.60 ± 10^{-2}	0.63 ± 10^{-2}	0.67 ± 10^{-6}	0.62 ± 10^{-2}
